# Substrate stabilisation and small structures in coral restoration: State of knowledge, and considerations for management and implementation

**DOI:** 10.1371/journal.pone.0240846

**Published:** 2020-10-27

**Authors:** Daniela M. Ceccarelli, Ian M. McLeod, Lisa Boström-Einarsson, Scott E. Bryan, Kathryn M. Chartrand, Michael J. Emslie, Mark T. Gibbs, Manuel Gonzalez Rivero, Margaux Y. Hein, Andrew Heyward, Tania M. Kenyon, Brett M. Lewis, Neil Mattocks, Maxine Newlands, Marie-Lise Schläppy, David J. Suggett, Line K. Bay

**Affiliations:** 1 Marine Ecology Consultant, Nelly Bay, QLD, Australia; 2 ARC Centre of Excellence for Coral Reef Studies, Townsville, QLD, Australia; 3 TropWATER (Centre for Tropical Water and Aquatic Ecosystem Research), James Cook University, Townsville, Queensland, Australia; 4 Lancaster Environment Centre, Lancaster University, Lancaster, United Kingdom; 5 School of Earth & Atmospheric Sciences, Queensland University of Technology, Brisbane, QLD, Australia; 6 Australian Institute of Marine Science, PMB 3 Townsville MC, Townsville, Queensland, Australia; 7 Division of Business Development, Queensland University of Technology, Brisbane, Queensland, Australia; 8 Australian Institute of Marine Science, Indian Ocean Marine Research Centre, University of Western Australia, Crawley, Western Australia, Australia; 9 Marine Spatial Ecology Lab, The University of Queensland, St. Lucia, Queensland, Australia; 10 Reef Joint Field Management Program, Great Barrier Reef Marine Park Authority, Townsville, Queensland, Australia; 11 School of Social Science, James Cook University, Townsville, Queensland, Australia; 12 Faculty of Engineering, Oceans Graduate School, The University of Western Australia, Crawley, WA, Australia; 13 Climate Change Cluster, University of Technology Sydney, Sydney, NSW, Australia; Uniwersytet Warszawski, POLAND

## Abstract

Coral reef ecosystems are under increasing pressure from local and regional stressors and a changing climate. Current management focuses on reducing stressors to allow for natural recovery, but in many areas where coral reefs are damaged, natural recovery can be restricted, delayed or interrupted because of unstable, unconsolidated coral fragments, or rubble. Rubble fields are a natural component of coral reefs, but repeated or high-magnitude disturbances can prevent natural cementation and consolidation processes, so that coral recruits fail to survive. A suite of interventions have been used to target this issue globally, such as using mesh to stabilise rubble, removing the rubble to reveal hard substrate and deploying rocks or other hard substrates over the rubble to facilitate recruit survival. Small, modular structures can be used at multiple scales, with or without attached coral fragments, to create structural complexity and settlement surfaces. However, these can introduce foreign materials to the reef, and a limited understanding of natural recovery processes exists for the potential of this type of active intervention to successfully restore local coral reef structure. This review synthesises available knowledge about the ecological role of coral rubble, natural coral recolonisation and recovery rates and the potential benefits and risks associated with active interventions in this rapidly evolving field. Fundamental knowledge gaps include baseline levels of rubble, the structural complexity of reef habitats in space and time, natural rubble consolidation processes and the risks associated with each intervention method. Any restoration intervention needs to be underpinned by risk assessment, and the decision to repair rubble fields must arise from an understanding of when and where unconsolidated substrate and lack of structure impair natural reef recovery and ecological function. Monitoring is necessary to ascertain the success or failure of the intervention and impacts of potential risks, but there is a strong need to specify desired outcomes, the spatial and temporal context and indicators to be measured. With a focus on the Great Barrier Reef, we synthesise the techniques, successes and failures associated with rubble stabilisation and the use of small structures, review monitoring methods and indicators, and provide recommendations to ensure that we learn from past projects.

## Introduction

The degradation of coral reefs worldwide is affecting the socio-ecological and economic value of reef ecosystems and the livelihoods of millions of people [1–3]. To date, coral reef management has focused on reducing local and regional stressors such as overfishing (through fisheries management and marine protected areas), coastal development (through permitting and mitigation), and improving water quality (through land and waste water management) to ensure reef health and facilitate natural recovery [4, 5]. More recently, there is acceptance that these approaches, even if enhanced, will not adequately protect coral reefs from the escalating effects of human use, and particularly from anthropogenic climate change [6–9]. An increasing awareness of the downward trajectory of coral reef condition has driven a search for additional active management activities, or interventions [9–11], to complement existing management and facilitate optimal conservation outcomes such as coral reef recovery and adaptation, at least at local scales [4, 12]. To date, coral reef restoration has been dominated by small-scale (10s to 100s of m^2^) projects using a few fast-growing species, with primarily aesthetic results, and their success or failure has been difficult to ascertain due to [13] a common lack of adequate monitoring [14]. Improving local conditions on small tracts of reef has had primarily aesthetic results, with little indication about their ability to restore reef communities, functions and processes. Practitioners are generally conscious that interventions and restoration efforts are not substitutes for global actions such as reducing greenhouse gas emissions, but it is hoped that they could provide a buffer, even at small and localized scales, between current conditions and a potentially improved situation in the future [9].

Much of the recent focus on reef degradation has concentrated on sources of coral mortality such as thermal bleaching [8]. However, an additional problem on many coral reefs is the creation of unconsolidated reef substrate or coral rubble through direct damage by storms and cyclones, dynamite fishing and vessel strike, and the increased fragmentation of coral skeletons following mortality after bleaching, crown-of-thorns starfish and disease outbreaks. Rubble fields are a natural part of the complex habitat mosaic of coral reefs, but can be associated with impoverished coral species assemblages [15–18] due to their instability and reduced structural complexity. Unattached rubble can act as a “killing field” for corals, inhibiting the survival of coral recruits and establishment of mature coral colonies [15, 17–23], and can cause further degradation by rolling and impacting remaining live coral colonies in high surge conditions [17].

**Fig 1:**
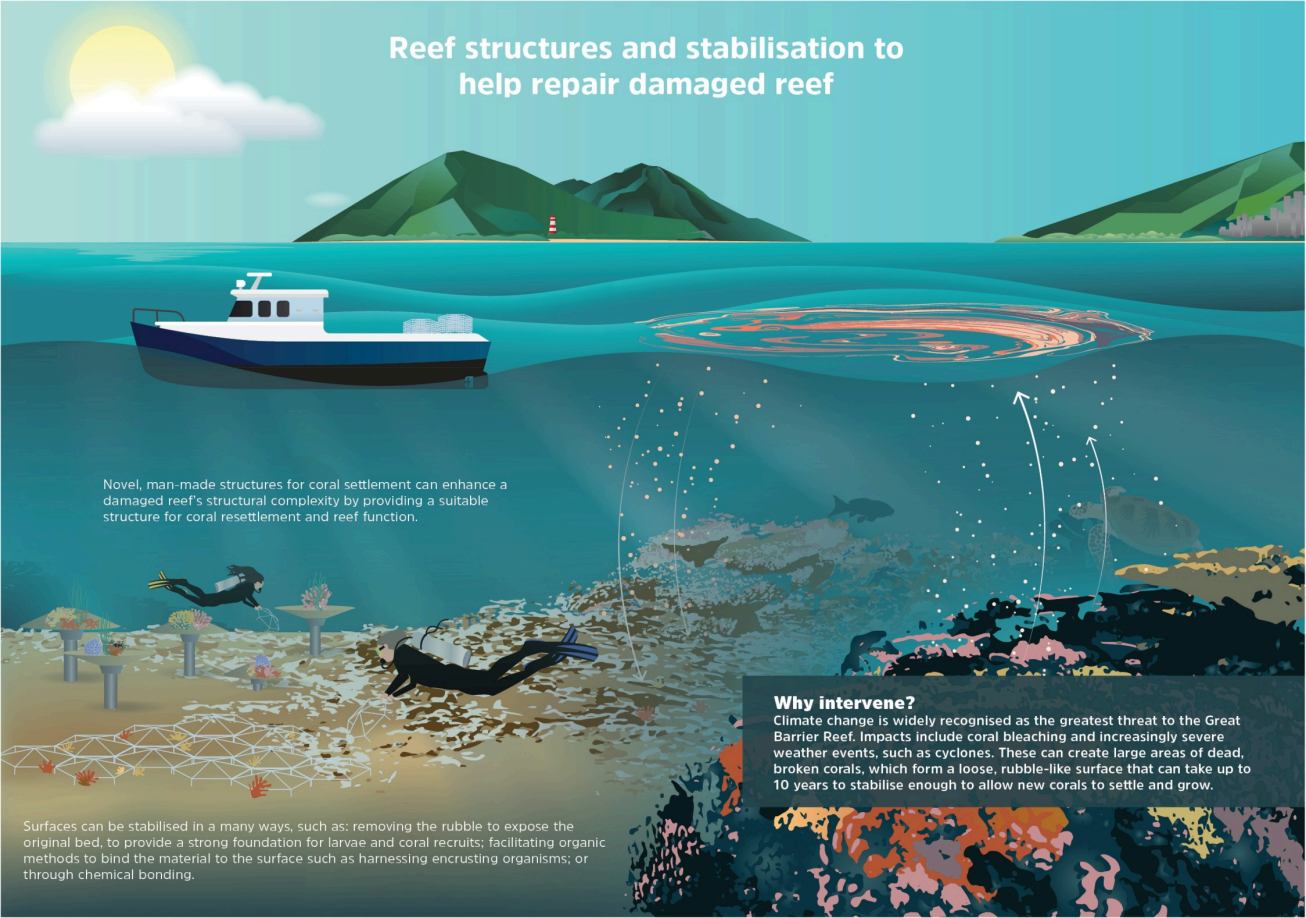
Reef structures to help stabilise damaged reef. Examples of structures used to stabilise and restore rubble-dominated habitats. Reproduced with permission of the Reef Restoration and Adaptation Program.

Rubble is often included as a benthic substrate category in coral reef assessment and monitoring programs. However, the extent and temporal dynamics of rubble are seldom quantified, despite sudden visible changes following disturbance events. When these dynamics are quantified, they are often excluded from reporting activities, precluding an indication of whether rubble fields are decreasing (e.g. due to consolidation and settlement by sessile organisms), increasing (e.g. due to repeated disturbance events and lack of recovery) or stable in time and space. A growing suite of interventions aims to assist coral recovery on rubble fields through various forms of substrate stabilisation (see examples in Fig 1). These range from simple rock piles placed on rubble fields to complex combinations of stabilisation techniques, specialised structures and coral transplantation.

Despite the growing promotion and use of many of these techniques, important knowledge gaps exist on the current extent of rubble fields and whether they are increasing, the drivers of rubble generation, natural processes of coral recovery in rubble fields, and the ‘point of no return’ when rubble fields are unlikely to revert to pre-existing habitats on their own. Because rubble fields created by disturbance events (e.g. ship strikes, blast fishing, cyclones) are often small (10s to 100s of m^2^) compared to other agents of coral reef damage, the potential exists for interventions to be successful in assisting coral recovery at this scale.

In this review, we:

- Explore the ecological role of loose coarse substrata (rubble) and structural complexity on coral reefs, and the capacity for natural coral reef recovery in destabilised areas
- Synthesise the techniques, successes and failures associated with active intervention to stabilise substrate and add small structures as coral restoration methods
- Review monitoring methods and indicators used to assess the results of these coral restoration practices
- Identify knowledge gaps and provide recommendations to ensure that we learn from a growing number of trials and projects.

We base our synthesis on peer-reviewed publications, reports, manuals and interviews on the importance of reef structural complexity and the effects of its breakdown into rubble, the processes involved in the natural stabilisation of rubble fields and return to stable reef matrix, and potential solutions being considered and implemented when natural recovery fails. We specifically review the use of small structures (from a few centimetres to ~2 m^3^) for active management interventions on coral reefs, and for the stabilisation of rubble. We exclude studies on artificial reefs [e.g. 24], which we define as artificial structures installed in areas where reefs have not previously existed, and approaches that rely on direct attachment of corals into the reef matrix, but refer interested readers to Boström-Einarsson et al. [14] where these methods are reviewed. We focus instead on studies that either add structures, or where corals are attached to frames or structures that are then placed onto, or attached to, damaged and unstable reef substrata.

We used the interactive online visualisation tool from Boström-Einarsson et al. [25] to extract publications describing substrate stabilisation and the use of small structures for coral restoration purposes. Global experts were also interviewed to gather first-hand information on methods typically used to stabilise rubble. Nine experts were identified through the authors’ networks and recruited for interviews through email invitations. The interviews were conducted over the phone, following a semi-structured format in which they were asked qualitative questions on the techniques being used for reef repair after a physical disturbance such as a storm or ship grounding (see full questionnaire in S1 Appendix). For the purpose of this study, we focused on responses specific to substrate stabilisation (Questions 3 3.2, S1 Appendix). Interviews with experts were conducted under the human ethics number H7799 granted by the James Cook University Human Research Ethics Committee. These data were used to define key knowledge gaps to guide future research and development, and implications for regulation and management.

## The structure and consolidation of reef substrata

### Importance of structural complexity on coral reefs

The structural complexity of a coral reef, primarily provided by scleractinian corals, is critical in supporting a biodiverse reef ecosystem and mediating ecological processes such as recruitment, predation and competition [26, 27]. For example, many coral reef fishes recruit as juveniles to coral colonies [28, 29], shelter amongst the branches [30, 31], and use them during reproduction [32]. High structural complexity on coral reefs has been correlated with low algal cover, high coral cover, and increased fish density and biomass [reviewed by 27]. The many microhabitats–crevices, overhangs and protrusions–characteristic of highly complex reefs can facilitate survival of organisms by affording places of refuge from predation [33–35], a broad range of environmental settings for water flow rates [36] and light [37], and by moderating density-dependent competition [38]. However, coral reefs are constantly undergoing destructive and constructive processes, which result in a natural balance between a range of microhabitats, from structurally-complex and high live coral cover habitats to rubble habitats of reduced 3-dimensional complexity. A better understanding of the principles of ecological succession on reefs following disturbances is crucial to improve the efficiency of conventional and new active intervention strategies [39–41].

### Ecological value of rubble fields

Rubble on coral reefs can be defined as dead coral skeleton or reef rock pieces that have fractured and been liberated by mechanical or chemical means and are larger in size fraction than sand (>2mm) [42]. Persistent fragmentation is normal in numerous scleractinian taxa [43], including massive and branching growth forms. Some species fragment year-round, while others are affected by episodes of particularly rough weather [44]. Direct formation of rubble via natural mechanisms generally occurs through mechanical breakage caused by wave action, storm swells and above-average wave conditions [45]. Indirect rubble formation occurs from dead and weakened standing coral skeletons following mortality of live tissue [45].

Disturbance-driven breakdown of structural complexity into rubble may lead to declines in some species. However, rubble can also support a unique suite of species. Biodiversity in cryptic microhabitats of rubble fields can be extremely high [116]. Coral rubble is rich in crypto-benthic fish species [46], a guild of species hypothesised to drive much of the productivity on coral reefs [47], and is the preferred habitat for a subset of coral reef fishes [e.g. damselfishes, 48]. Coral rubble contains an abundance of crustaceans over three orders of magnitude higher than live coral branches [49]. Rubble is also an important habitat for free-living corals [50, 51] which, together with the rubble itself, can be important building blocks in reef accretion [42]. Further, rubble has a sedimentological role, providing the raw material for finer (carbonate) sediment that infills inner reef and lagoonal areas, creating new and different habitats, as well as cay islands (either rubble ramparts or sand islands).

Rubble is also formed by anthropogenic disturbances, including trampling, boat anchoring, dynamite fishing, coral mining and ship groundings [42, 52–55]. Many of these disturbances are expected to increase in frequency in the future, shrinking the window available for recovery [56, 57]. There is concern that current disturbance rates are converting living coral reefs into rubble fields at a rate exceeding the natural capacity for coral reef ecosystems to recover naturally [58, 59]. Coral reef systems around the world, such as the Greater Barrier Reef [8] and in Hawai’i [60] have already experienced recurrent cyclones and bleaching events in the past four years. As a result, the ratio of dead rubble to live coral cover is expected to increase, potentially slowing coral recovery.

### Natural stabilisation of rubble

**Fig 2:**
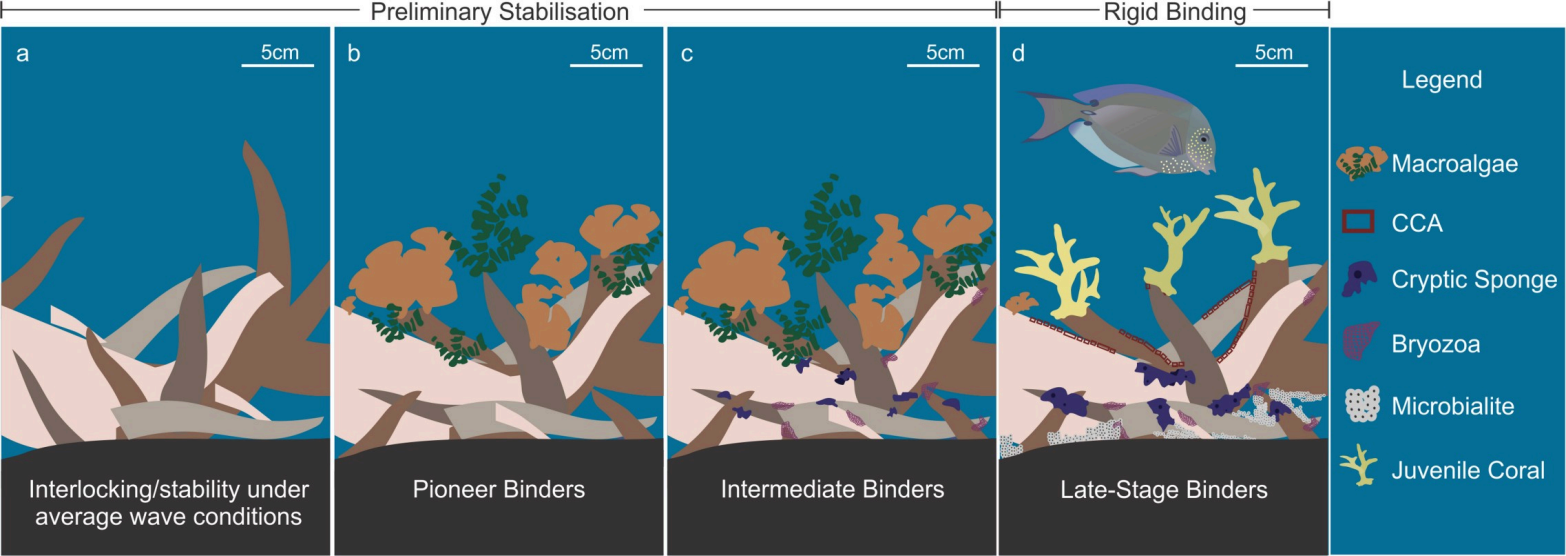
Stages in the stabilisation and binding of rubble. Stages of the natural stabilisation of rubble fields and eventual conversion to reef framework. A) shelter from strong hydrodynamic activities, a depression in bathymetry or particle organisation into stable bedforms allows the rubble pieces to settle, interlock and stabilise; b) pioneer binding organisms such as fleshy and calcareous algae settle on the rubble; c) intermediate binders such as cryptic and erect sponges create greater stability; and d) late stage binders and coral settlement.

If rubble remains stable for long enough, it can eventually be consolidated and incorporated into the reef framework. Reef cores are sometimes composed of layers of both unconsolidated and consolidated rubble, laid down over thousands of years of reef erosion and accretion [61, 62]. The transition from rubble to reef has been described as a two-part process that requires both a preliminary stage of rubble stabilisation and binding, followed by rigid binding and diagenetic cementation (see S2 Appendix for detail). Though this is a critical process in the recovery of damaged coral reef communities, our understanding of rubble stabilisation and binding rates, and the characteristics indicative of adequately bound and stabilised rubble, is limited [63]. Preliminary stabilisation of rubble can be achieved when hydrodynamic energy is reduced, rubble pieces interlock or attain a stable configuration (Fig 2A), followed by the colonisation of macroalgae, algal turfs and encrusting and binding invertebrates such as cryptic sponges (Fig 2B and 2C) [20, 21]. Using settlement tiles, Doropoulos et al. [64] established that the succession of organisms from turf algae to coralline algae can take at least six months depending on microhabitat, and Wulff [21] highlighted that rubble seeded by cryptic sponges could undergo stabilisation and colonisation by corals within 10 months. Despite these studies, the rates and characteristics that define when preliminary stabilisation is completed are poorly understood. Following preliminary stabilisation, rigid binding by laterally-growing carbonate encrusting organisms and diagenetic cementation can then occur (Fig 2D).

The reef framework is continuously infilled with sediment, rubble and cements, as part of diagenetic alteration [42, 65, 66]. These cements are largely composed of high‐magnesium calcite and aragonite that can infill the voids between the rubble pieces and encrust over the dead skeletal materials [61, 67]. Calcifying microbialites further trap and bind sediments and are important in providing a rigid cementation (S2 Appendix). The highest rates of rubble cementation are found in fore-reef areas with low sloping angles above the wave base, while the lowest rates are found in deeper fore-reef environments [42].

### When rubble fails to stabilise naturally

**Fig 3:**
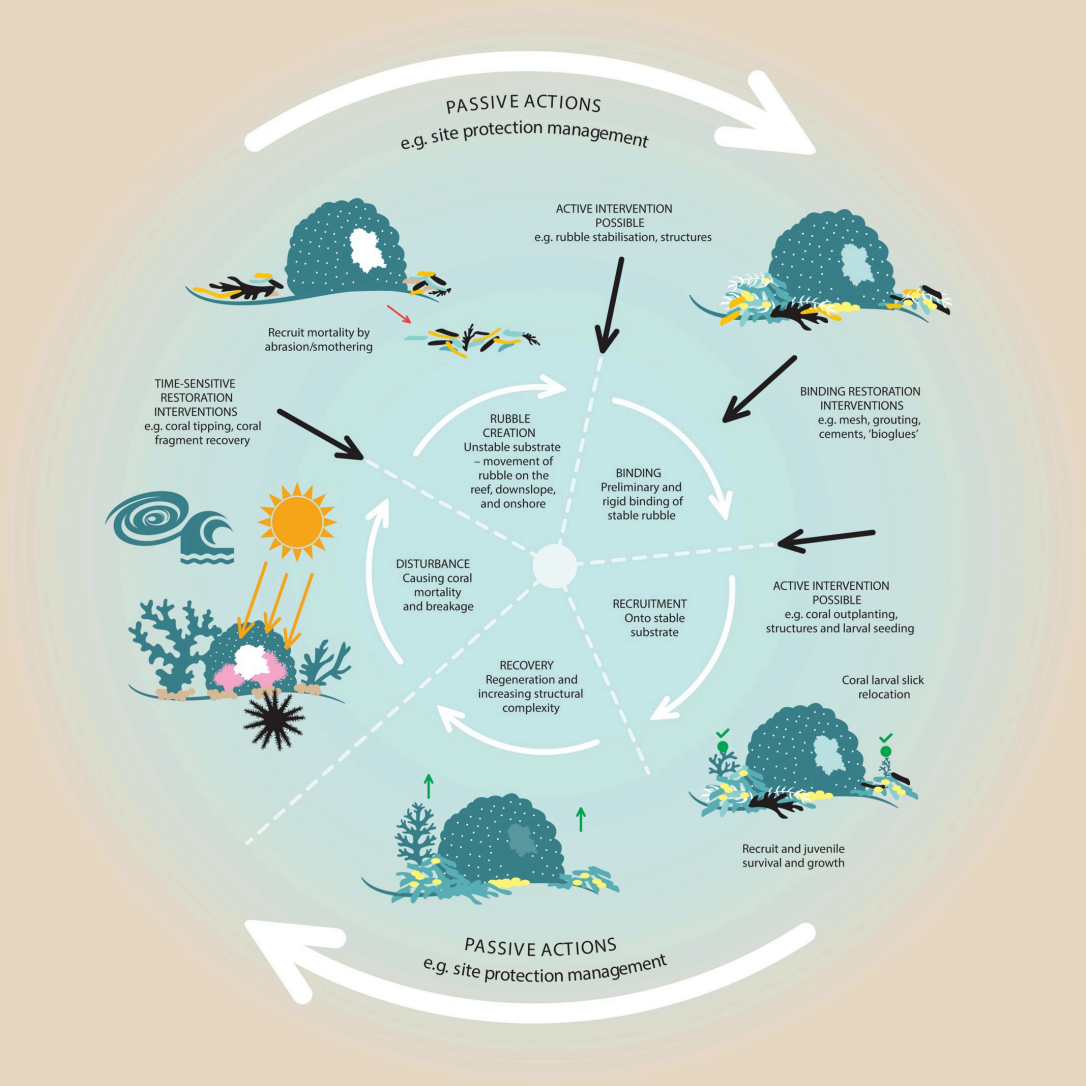
Where does active intervention fit into the disturbance and recovery cycle?. Schematic diagram showing formation of coral rubble, and stages that might require intervention. In the inner ring of the circular progression, the substratum is affected by disturbance, and, given favourable conditions, transitions from loose rubble to stable reef matrix onto which corals (outer ring) can recruit. When the transition from rubble creation to binding cannot occur naturally, it can be artificially induced through active restoration. Further intervention is possible through the seeding of coral larvae or attachment of coral fragments. When the cycle occurs naturally, passive management (e.g. through protected areas) can occur.

Once a rubble field is formed, the attachment of new coral larvae and their subsequent survival in the rubble as recruits depends primarily on the stability of the rubble [22, 68]. Lack of recovery of corals in rubble fields following disturbances such as dynamite fishing and ship groundings has been linked to rubble instability and hydrodynamically-driven rubble movement. This prevents the natural stabilisation, binding and cementation processes described above. For example, rubble pieces in dynamite-fished rubble beds at a depth of 6–10 m in Indonesia moved by up to 50 cm per day [22]. At a ship-grounding site in the Caribbean, pieces in the rubble bed at a depth of 9–14 m were estimated to be overturned every 12 days, and every 3.8 days during peak months [18]. Seventeen years after damage from dynamite fishing ceased, the rubble beds in Indonesia displayed significantly lower coral cover than rehabilitated and control (not blasted) reef sites, despite an adequate supply of coral larvae [S3 Appendix; 15, 69]. Coral larvae can, in fact, recruit to unconsolidated rubble, but frequent movement results in high mortality and impaired reef recovery [17]. Unconsolidated coral rubble also provides a poor substrate for asexual coral reproduction and growth [68, 70], although naturally-generated fragments from some species of *Montipora* and *Acropora* have been shown to persist when natural rubble zones remain stable [44, 71]. Thus, any restoration project using coral outplanting methods in damaged areas of unconsolidated rubble may benefit from preliminary substrate stabilisation (Fig 3). Consideration of when and where to implement these interventions, however, relies on a detailed understanding–building on the studies described above–of the threshold hydrodynamic conditions that cause rubble movement and impaired rubble field recovery.

The ratio of dead rubble to live coral cover is expected to increase as disturbances such as storms, cyclones and warming events leading to coral bleaching become more frequent, and intense, and degradation of reefs generally increases worldwide [56, 72, 73]. However, although ‘rubble’ is a commonly measured substrate category and proposed indicator for reef health [74, 75], rubble cover is often underreported in comparison to live coral cover, and there are currently no large-scale data sets showing changes in the proportion of rubble cover to consolidated reef. Several studies show the persistence of rubble fields that were created by disturbance or damage. In Indonesia, impacted sites at 6–12 m depth that were affected by disturbances including overfishing, pollution, blast fishing, trampling, anchor and boat damage, had 10–30% rubble cover, compared to <2% at control sites with similar wave exposure [76]. In Ecuador, sites impacted by overfishing, anchoring and derelict fishing gear had ~10–25% cover of rubble, and on the reef crest the percentage of rubble increased with the amount of derelict fishing gear [77]. In the Maldives, eight years after the 1998 bleaching event that caused mass coral mortality, rubble and sediment cover was still high, ranging from 15 to 65% [78], consistent with the reported losses in three-dimensional structural complexity [79]. This was also the case in the Seychelles, where some sites were still rubble-dominated (72% cover) in 2010 after the 1998 bleaching event, and these sites had significantly less cover of adult corals (10%) compared to sites on consolidated reef (33%) [80]. Following the ‘Kona’ storms in Hawaii in 1980, *Porites compressa* thickets on the reef slope were reduced to rubble of < 5 cm, and 12 years later there was only a small increase in the cover of *P*. *compressa* (0.5 to 1.5%). Rubble accumulations were sometimes moved downslope, but appeared to remain unconsolidated and subject to movement [81].

## Active intervention to stabilise substrate and add structure

### Substrate stabilisation

**Fig 4:**
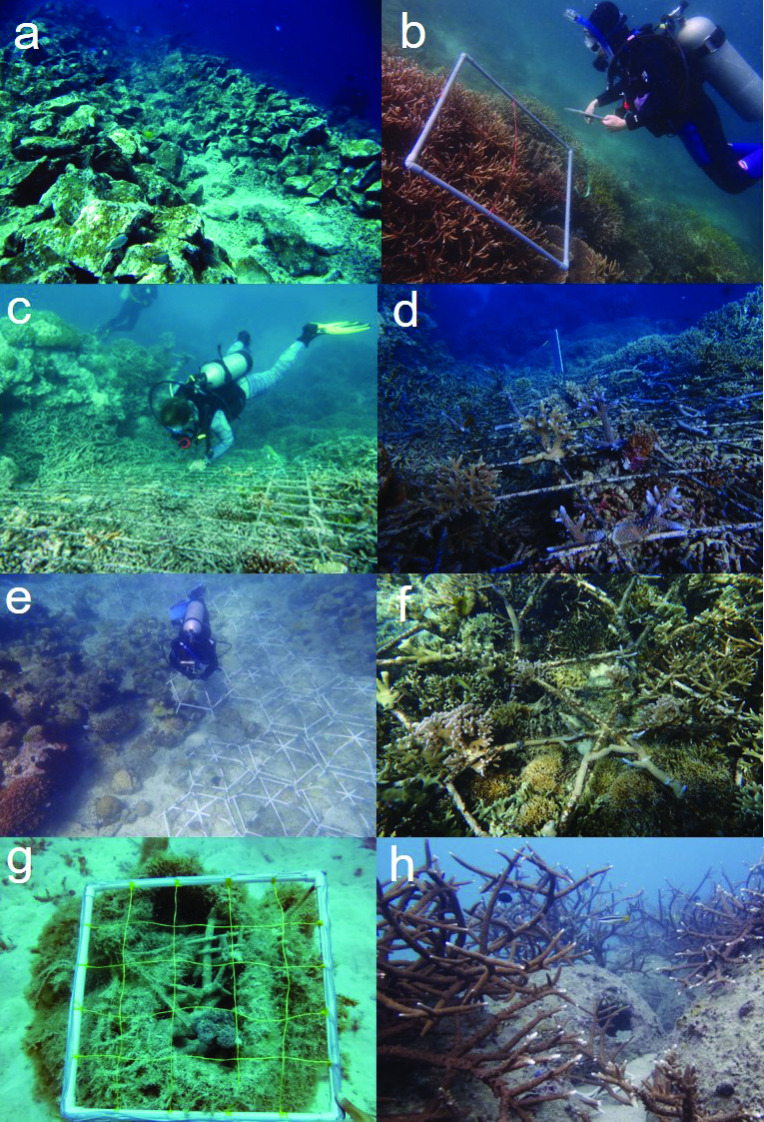
Methods and structures for rehabilitation of rubble fields. Rubble stabilisation techniques: (a) rocks used to consolidate rubble in Indonesia, photo by Helen Fox, (b) the same area 14 years later, photo by Emily Darling, (c) metal mesh used to stabilise rubble in Australia, photo by Ian McLeod, (d) the same mesh with corals added, photo by Nathan Cook. (e) Reef Stars deployed over a rubble bed in Indonesia, photo by Biopixel. (f) Reef Stars with coral growth, Indonesia, photo by Marie-Lise Schlappy, (g) reef bags used to consolidate rubble in Australia, photo by Tom Baldock, (h) corals growing on reef balls in Thailand, photo by Margaux Hein. Note that many of these methods have not been subject to rigorous scientific testing for effectiveness, and are shown here as examples.

Physical restoration of mechanically-damaged coral reef areas has been relatively common in US territorial waters, often funded by insurance claims following ship-strikes. The most common technique has been to remove the rubble and re-plant the dislodged coral colonies using cement; this method has been used both after ship groundings and following the 2004 tsunami in southeast Asia [e.g. 82, 83]. Also common is the installation of mesh or netting over the rubble to prevent further movement and encourage natural binding and cementation processes [Fig 4 and Table 1; 10, 84]. This is generally a precursor to transplanting corals or deploying artificial structures onto the damaged area [68]. Other methods include driving metal reinforcement bars into rubble (see S3 Appendix for case study) [85], piling large rocks onto unstable degraded reef areas [69], collecting rubble into open cement structures [54] or natural fibre (sisal) net bags and placing them on the damaged reef, or injecting grout or other chemicals to bond and stabilise loose rubble [86]. Implementing these approaches requires assessing the benefits of substrate stabilisation in the context of the natural potential for consolidation on a particular reef. Such stabilisation activities may be beneficial on a small scale at high-value sites [85], or following ship groundings that generate expansive areas of unconsolidated rubble on a previously well-consolidated reef framework [87].

**Table 1:** Rubble stabilisation and small structures: Summary of methods.

Approach	Primary goal	Advantages	Disadvantages	Outcomes (References)
Coral tipping (replacing overturned corals after mechanical damage)	Enhancing survival of overturned corals	• No extraneous materials needed	• Small scale	• Large *Porites* colonies thrown onto land by a storm and replaced into subtidal reef. ***Positive outcomes***: abundant recruitment and increase in fish abundance using the colonies. ***Negative outcomes***: months between impact and intervention killed most of the coral tissue, high cost and machinery required, original coral tissue died [103]. Considered a temporary measure to precede the use of cement [83].
		• Negligible to no material costs	• Must occur rapidly after disturbance	
		• Promotes natural processes of attachment and survival		
			• Ineffective in naturally high-energy environments	
		• Retains existing habitat structure		
			• If colonies are large (e.g. *Porites*), heavy machinery may be required	
			• Subject to movement during storms	
			• Manual activity, potentially high labour cost	
Coral reattachment	Use of cement to attach individual storm-blown colonies to enhance survival	• Negligible to no material costs	• Small scale	• ***Positive outcomes***: Successful attachment and low mortality of colonies, species composition similar to pre-disturbance [82].
		• Promotes natural processes of attachment and survival	• Must occur rapidly after disturbance	
			• Must be preceded by rubble removal (see below)	
		• Use of original coral assemblage		
			• Some machinery required (cement mixer)	
			• Depends on rapid setting of cement	
			• Subject to movement during storms	
			• Manual activity, potentially high labour cost	
Rubble removal on reef slopes and flats	Exposing solid substrate underneath, to encourage settlement of sessile organisms	• Does not impact reef aesthetics	• Small scale	• Removal of rubble after ship grounding. ***Positive outcomes*:** Successful removal of rubble after a number of attempts and engineering problems. Exposed bare consolidated substratum for coral reattachment (S3 Appendix).
		• Allows attachment and settlement of corals onto exposed solid substrate	• Potential negative impact at rubble disposal site if offshore	
			• Death of organisms living in/on rubble	
			• Does not add structural complexity	
			• High cost	
Metal stakes	Provision of settlement substrate	• Cheap materials, readily sourced locally	• Small scale	• No literature to assess outcomes
			• Limited potential to trap and stabilise unconsolidated substrata	
		• Becomes inconspicuous relatively quickly (gaining aesthetic appeal)		
			• Unknown how microbiome may be affected by materials, and how this might affect recolonisation success	
		• Quick and easy deployment–does not require complex machinery		
			• May act as habitat for unwanted organisms	
			• Introduction of foreign material	
Metal stakes and plastic mesh netting	Substrate stabilisation and provision of settlement substrate	• Cheap materials, can be sourced locally	• Small scale	• ***Positive outcomes***: Increase in fish biomass, coral recruit size and coral recruit survival (63% vs 6%) after two years ***Absent outcomes***: Non-significant increase in coral cover. ***Negative outcomes***: plastic netting still visible after 5 years [84] (S3 Appendix).
			• Microbiome may be affected by materials, potentially limiting recolonisation success	
		• May become inconspicuous (gaining aesthetic appeal)		
		• Quick and easy deployment–does not require complex machinery	• May act as habitat for unwanted organisms	
			• Likely restricted to relatively sheltered areas for deployment success and long-term stability	
		• Corals known to settle on both stakes and netting		
			• Risk of burial by surrounding rubble during storms due to low profile	
		• Prevents movement of loose rubble		
			• Introduction of foreign material	
			• Use of plastic for netting can introduce debris once breakdown begins	
Inject chemicals (usually cement) to bond unconsolidated substrates	Substrate stabilisation	• Often cheap materials, readily sourced locally	• Diffuse deployment (potential to contaminate non-degraded areas)	• No literature to assess outcomes
		• Can be deployed over moderately large areas (10–100 m^2^) with little expertise	• Difficult to do underwater.	
			• Unknown toxicity of chemicals to rubble biota and other organisms	
			• Likely restricted to relatively sheltered areas for success	
		• Speeds the consolidation of rubble fields towards suitable settlement substrata		
3D frames (e.g. MARRS Reef Stars)	Substrate stabilisation and providing habitat structure	• Modular, ready scope to scale (quick and easy deployment–does not require complex machinery)	• Potential refuge of corallivores, hindering coral recruit survival	• ***Positive outcomes***: MARRS Reef Stars resulted in increase in coral cover from 10% to over 50% after three years [99] (S3 Appendix).
			• May require further ecosystem modification to establish (e.g. damselfish/corallivore removal)	
		• Can trap unconsolidated rubble from adjoining degraded reef areas		
			• Reef stars must be sourced from supplier and involves cost for bespoke fabrications (under patent).	
		• Can provide improved growing conditions for coral (higher than surrounding benthos)		
			• Addition of structures may incur high permitting risk	
			• Unknown resistance to high hydrodynamic energy	
		• Becomes inconspicuous relatively quickly (gaining aesthetic appeal)•		
			• Adding plastic, epoxy and steel to the marine environment	
			• Microbiome may be affected by materials, potentially limiting	
			• recolonisation success	
			• May act as habitat for unwanted organisms	
		• Provide/facilitate refuge for fish and invertebrates		
			• May serve as fish attracting devices, drawing fish from natural habitats	
		• Can be fixed in place or temporary for removal		
			• Visible for several months, reducing aesthetic appeal until the coral covers the frames	
		• Installation can allow for strong community engagement		
BioRock^TM^; mesh frames (with or without electrical current)	Substrate stabilisation and providing habitat structure	• Same as for 3D frames; also:	• Same as for 3D frames; also:	• ***Positive outcomes***: Increased attachment rates, survival and / or growth of coral fragments [97, 108–112], densities of reef associated fishes 6 times greater [113]. ***Negative outcomes***: Decreased growth of fragments [114]. ***Absent outcomes***: No change in growth rates [100].
		• Potential for facilitating/increasing levels of cementation within the rubble bed	• Requires source of power adding costs and logistical challenges	
			• Current required for many months for good accretion	
		• Eventually incorporated into the reef framework		
SECORE Tetrapods	Providing structure for coral recruitment	• Relatively inexpensive materials, readily sourced locally	• Small size reduces scalability	• ***Positive outcomes***: 5 to 18-fold reduction in out planting costs compared to direct methods. ***Negative outcomes***: low survivorship of coral recruits, rapid colonization by algae [98].
			• Need to be wedged into complex reef structure; role in	
			• rubble is unclear	
		• Can be deployed by divers	• Introduction of foreign material	
		• May resemble consolidated reef substrate with aesthetic appeal		
			• Colonization by undesired organisms	
		• Eventually incorporated into the reef framework	• High labour (diver) costs	
Natural or concrete-fabricated structures: Reef Balls^TM^; Subcon reef modules; boulders, pipes and large objects.	Substrate stabilisation and providing habitat structure	• Modular design facilitates scalability	• Larger scale habitat engineering may incur high permitting risk	• ***Positive outcomes***: Reef Balls^TM^ provide shoreline protection and lead to increased fish abundance [115, 116]. ***Negative outcomes***: Low coral recruitment [116].
		• Often relatively inexpensive materials, readily sourced locally (except Reef Balls^TM^)	• Ecological (and climatic/biogeochemical) impacts of different grades of concrete	
				• ***Positive outcomes***: Subcon modules were colonised by invertebrate and fish fauna similar to a nearby shipwreck in 20 months. ***Negative outcomes***: The modules were rapidly colonised by algae [106].
		• Can create habitat structure at scale easily	• Patented structures must be sourced from supplier and involves cost for bespoke fabrications (under patent).	
		• Promotes biodiversity, and can withstand some physical stress as scale increases		
			• Installations increasingly permanent as scale increases	
				• ***Positive outcomes***: Tubular pipes completely overgrown with *Porites* colonies in 12 years. ***Negative outcomes***: The *Porites*-dominated community replaced assemblages originally composed of *Acropora* thickets [117].
		• Provide/facilitate refuge for fish and invertebrates		
		• May resemble consolidated reef substrate with aesthetic appeal	• Almost always requires heavy machinery	
			• Introduction of foreign material	
		• Eventually incorporated into the reef framework, depending on size	• High risk of sedimentation onto colonised substrate in areas of degraded reef	• ***Positive outcomes***: Rock piles resulted in increase in fish communities similar to those of healthy reefs, hard coral cover from 0% to 44.5% over 14 years [69], (S3 Appendix).
		• Reef Balls^TM^ moulds can be bought from the company for different sized structures and fabricated on site using locally sourced cement plus admixtures	• Microbiome may be affected by materials, potentially limiting recolonisation success	
			• May act as habitat for unwanted organisms	
			• May serve as fish attracting devices, drawing fish from natural habitats	
			• Sustainability issues around concrete production and transportation	
Gabion cages/baskets/reef bags	Substrate stabilisation and providing habitat structure	• Mostly the same as for ‘natural or concrete-fabricated structures’–accessible and relatively low cost	• Mostly the same as for ‘natural or concrete-fabricated structures’ except:	• ***Positive outcomes***: Reef bags stable, CCA recruitment, increased fish abundance, some coral recruitment after 7 months [104].
			• May require heavy machinery	
		• Filled with existing natural materials (e.g. reef rubble primed for coral recruitment)		
		• Eventually incorporated into the reef framework		
		• Can be constructed *in situ* by divers		
		• Provide shoreline protection if designed and positioned correctly		

The lack of documentation on substrate stabilisation suggests that this is an area where logistics and materials are still under development [14]. In addition to ship strikes and dynamite fishing, natural disturbances such as storms and cyclones contribute to the types of impacts that convert complex coral cover to shifting rubble fields that may or may not consolidate over time [17]. Studies on coral survival on degradable structures caution that materials need to be sufficiently durable [88]. This highlights one of the risks of investing time and resources in substrate stabilisation, where increasing storm activity may damage or dislodge structures.

### Small structures

Beyond the simple stabilisation of rubble, the deployment of small structures (from a few centimetres to ~2 m^3^) aims to either combine substrate stabilisation with the provision of habitat structure, or to provide structure only (Table 1). This is one of the earliest forms of coral restoration [14]. Early attempts often involved using discarded objects like car tires and steel frames to rapidly create structural complexity in degraded habitats [89, 90] (but see Risks section). However, in recent years, objectives and methodology have shifted towards engineered structures that mimic specific functions performed by an intact coral reef framework [Fig 4; 91]. The most commonly-used structures are frames [92, 93], blocks [91], rocks [69, 84] or purpose-built structures such as ReefBalls^TM^ [94], ceramic EcoReefs [95], BioRock [96, 97], SECORE (Sexual Coral Reproduction) tetrapods [98] or MARRS (Mars Assisted Reef Restoration System) Reef Stars (previously known as ‘Spiders’) [99]. Three-dimensional habitat can be enhanced by designing novel structures for settlement [e.g. new shapes, sizes and surfaces; 96, 98], or by using electrical current to stimulate mineral accretion [97, 100, 101], with or without the attachment of coral colonies or fragments. Given adequate upstream larval sources, successful structures can attract natural coral recruitment and enhance survival or provide habitat for other reef organisms [102].

There is very little evidence, and in some cases, none at all, of the success or failure rates of the different methods (Table 1). Positive outcomes of methods used to date include coral recruitment onto the structures (overturned corals and reef bags [103, 104]), increasing coral cover on and around the structures (MARRS Reef Stars, rock piles [69, 99]) and increasing fish abundance and biomass (metal stakes, ReefBalls, Subcon modules [84, 105, 106]). However, there were similar numbers of studies reporting failed coral recruitment due to algal overgrowth of the structures [98], no change in coral cover, or artificial structures remaining visible before the end of the monitoring period [84]. Some methods required a period of trial and error before they could be deemed useful (e.g. rubble removal from a ship grounding site, S3 Appendix), and others resulted in structures remaining visible, rather than becoming incorporated into the reef matrix. For some methods, such as BioRock, results reported by different researchers are equivocal, with some reporting increased attachment rates and others finding no difference (Table 1). No literature presently exists describing efforts to use metal stakes for rubble stabilisation, or to use cement or similar chemicals to enhance rubble stabilisation rates. Studies reporting positive outcomes were usually conducted over a short time-frame, preventing an assessment of whether there was, in fact, a restoration of coral communities and processes at any scale (Table 1).

**Fig 5:**
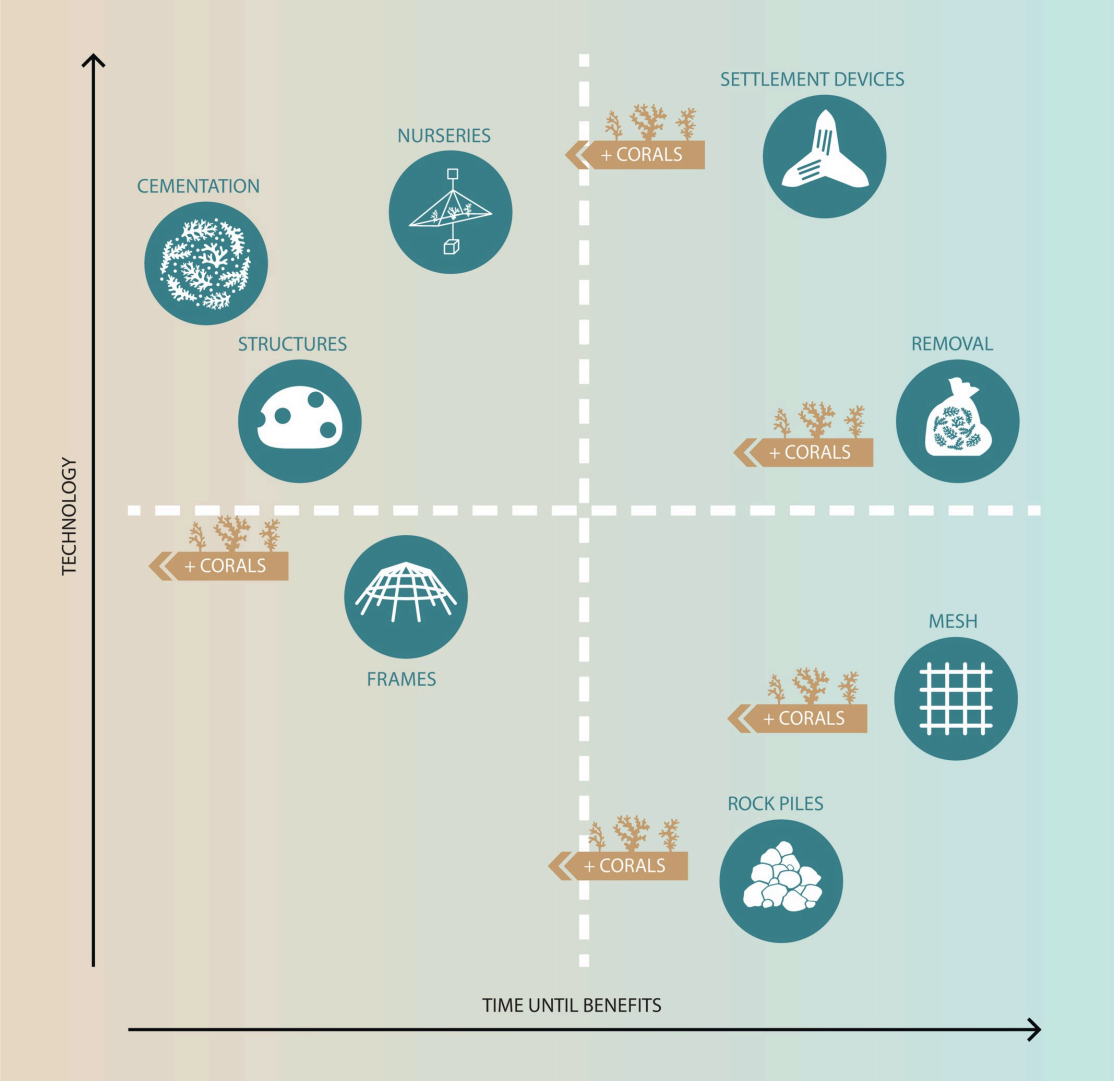
Costs and benefits of rubble field restoration methods. A stylised visual representation of the relationship between time required to gain restoration benefits and the level of technology required. Techniques in the top left quadrant require a higher level of technology, but are likely to yield immediate benefits. Techniques in the top right quadrant are more technologically advanced and will take a relatively long time before recovery occurs (i.e. through natural recruitment and growth of corals). Techniques in the bottom right quadrant are relatively low-tech but are expected to take a long time to yield benefits. Finally, techniques in the bottom left are low-tech and may see immediate or fast benefits to coral communities. Most techniques can reduce the time until benefits (moving right to left along x-axis) by adding transplanted corals.

Theoretically, the time required for restoration techniques to benefit coral assemblages at scales of 10s to 100s of m^2^ (e.g. increased fish abundance, coral cover, structural complexity) depends to some degree on the technology and mechanisms used (Fig 5). Techniques such as deploying tailored structures require a high level of technological investment and man-power, but are expected to yield immediate benefits by providing shelter and structural complexity for sessile organisms and reef fishes. Deploying cementation compounds and removing rubble are also technologically advanced but are likely to take variable amounts of time before recovery occurs through natural recruitment and growth of corals. Some low-tech methods, such as mesh or rock piles to stabilise loose rubble, would also take a long time to translate into benefits on the reef through coral growth, while others such as frames that provide shelter and structure could yield immediate results in terms of fish abundance and diversity, although this may occur through novel structures attracting fishes from natural habitats nearby. The time to yield results can be sped up for most techniques by transplanting corals onto the artificial structures (Fig 5).

### Benefits

The provision of stable substrata is primarily of benefit to corals and other sessile organisms, which can settle and grow without the risk of being overturned, broken, abraded or smothered as loose rubble moves around [107]. Once sessile organisms, particularly corals, have become established, their growth provides further three-dimensional habitat for motile organisms [99]. The methods described above can, in some cases, be integrated and used together, and ideally become part of the reef framework over time; documented projects include examples where this occurred and others where it did not (Table 1). Practically, small and modular structures are relatively easy to fabricate and handle, have a low per-unit cost, can be quickly deployed, and can be less complex, from a regulatory and permitting perspective, than larger structures such as artificial reefs (S3 Appendix).

Summary of advantages, disadvantages, successes or failures of different approaches (from simple to more complex) to the use of structures and substrate stabilisation.

Although financial profit is not generally the primary motivation of restoration practitioners, sociocultural and economic indicators for coral restoration effectiveness include reef users’ satisfaction, stewardship, capacity-building, and economic value [118]. Substrate stabilisation and the use of small structures can therefore have economic benefits through enhancing recreational diving, fishing and tourism at new or recovering sites, especially when using structures that enhance overall habitat complexity [119]. Such sites may act to reduce pressure on natural reefs, allowing disturbed reefs to recover with reduced damage from snorkelers and/or scuba divers. Increasing fish stocks and recreation markets can benefit local communities through food security and economic opportunities [85].

### Risks

The effects of substrate stabilisation and structure deployment should be considered within a risk framework [120] to further guide coral restoration regulation, research and development. Undesirable outcomes could include the potential shifts in coral community composition due to novel substrata favouring some species over others [119], as well as impacts on rubble habitat biota [46, 47] and the overall carbonate sediment production of the reef. If not prepared correctly, artificial structures could introduce pathogens, and poor placement may result in movement, damage to adjacent areas, or loss (Table 1). Often, it is assumed that small structures or mesh netting will become part of the reef framework over time, but if materials are inappropriate (e.g. Raymundo et al. [84] and Fadli [121] used plastic netting) there is a risk of degradation, generating marine debris and contaminating the marine environment [14]. Consequently, the location and desired scale of deployment needs to be considered, as an optimum restoration may not require intervention over an entire rubble bank.

Poor placement can result in smothering sessile organisms, and if structures are used as a vector for introducing coral fragments, the question of the source of those fragments arises [122]. There has been some research to suggest maximum ‘safe’ proportions of healthy *Acropora cervicornis* and *Stylophora pistillata* colonies (10% or less) that can be harvested for fragments [123, 124], but further research needs to take into account a broader range of species in a variety of environmental settings. If collecting ‘fragments of opportunity’, practitioners need to ensure that they are not denuding an area of coral fragments that may have otherwise become attached and survived to produce new growth at the source location. Risks of failure in delivering a successful intervention will also depend on understanding the dynamics of rubble transportation within a reef, to identify suitable areas and/or techniques of intervention that consider the regular deposition of rubble naturally produced in a reef system. Substrate stability is likely to be naturally patchy even at small spatial scales, with rubble accumulating in depressions and limestone or live coral outcrops providing raised consolidated patches. There is still uncertainty about our ability to make an ecologically meaningful difference in attempting to restore denuded consolidated substrate; one management option is to focus on firstly restoring this type of habitat, before resources are allocated to rubble.

While the temptation to undertake interventions is sometimes understandable, stabilising rubble and establishing manufactured structures can fail at a higher rate than restoration projects on consolidated reef, due to unfavourable environmental conditions which precluded coral growth in the first place [122]. This includes the concern that increasing frequency and intensity of disturbances could potentially undo the restoration work that has been implemented; there is a risk that significant resources and effort channelled into restoration projects that may be negated by the next disturbance. Physical modelling is one tool that can ensure that restoration structures can withstand rough storm conditions. For example, ‘reef bags’–netted bags filled with rubble–were made to specifications likely to withstand velocities experienced during violent storms (Fig 4G). To minimise risk in future projects, the publication of failures in coral restoration is at least as important as publishing success stories. This would greatly assist cost-benefit analyses to support future decisions about restoration methods, as would more detailed information about the costs of interventions. Importantly, the risk of taking action also needs to be weighed against the risk of doing nothing, and the potential consequences of allowing reefs or sections of reefs to degrade completely.

Limited government support and funding, a need for new or refined policy, plans relevant to restoration and adaptation [125], better enforcement and reduction in permitting constraints (see S4 Appendix) have all been identified as general limitations to coral restoration effectiveness [126]. While substrate stabilisation has been identified as a potentially useful technique, it has not been a focus in reef intervention policy, especially in Australia (S4 Appendix). Further, it is only prioritised in the legal framework of responses to damage caused by ship groundings or storms in some parts of the world (e.g. the US and the Caribbean). Approval processes for specific restoration projects are generally dependent on the level of risk and location. Regulatory permitting will range from low risk (substrate stabilisation) to high risk (habitat engineering and artificial reefs), depending on the specific delivery methods used. Additional studies are required to determine optimum material types, shape or configuration and orientation, season of deployment, and the specific benefits and risks associated with each.

### Scaling-up

Given the substantial and increasing area of coral reefs that may require restoration or rehabilitation, and the inherently small scales of restoration activities presently being practised, current practices would need to be scaled up by many orders of magnitude to demonstrably slow down the global and regional rate of reef degradation. To be effective at regional or larger scales, existing substrate stabilisation techniques would need to be replicated many millions of times (Table 1).

There are two approaches for scaling up. First, current methods can simply be replicated, similar to the development of the global agriculture or forestry sectors [127, 128]. However, few opportunities exist for gaining economies of scale by simply applying the same technique repeatedly; additionally, the patchiness of the habitat and depth-related variability are important differences between coral reef environments and terrestrial habitats. A second approach focuses on methodological changes and/or the development and uptake of new technology to achieve major per-unit cost reductions [127]. This second approach, also exemplified through global agriculture, aims to increase efficiency and productivity by developing and implementing biological and mechanical technology. There are increasing efforts to develop technology through better understanding of biological and ecological processes–such as natural stabilisation rates and rubble movement hydrodynamic thresholds. These will likely lead to some gains in productivity, for example knowing exactly when and where to carry out interventions. However, scaling-up many, if not most, of the current practices will not result in substantial per-unit efficiencies unless mechanisation and automation can be developed and applied. Therefore, scaling-up approaches needs to be explicitly considered when planning method development, pilot projects and research programs.

## Monitoring and measuring success

Coral reef restoration and adaptation projects would benefit from knowledge of the baseline (pre-disturbance) state, and an effective monitoring program to assess the performance of intervention methods. This evaluation should be based on a clear statement of objectives, translated into appropriate indicators or metrics to be measured, using adequate sampling techniques, time frames and spatial scales [13]. When considering substrate stabilisation and small structures, initial, short-term metrics may include measures of substrate stability followed by long-term assessment of changes in coral cover and the ratio of coral to rubble, recruitment, assemblage structure and structural complexity (Table 2). Overinvestment in stabilisation can also impact on the natural sediment budgets of coral reefs, so an understanding of the background rubble state of reefs is important. Other metrics may relate to broader ecological function as suggested by Hein et al. [118], including coral diversity, herbivore biomass and diversity, and coral health. All these metrics can be monitored using well-established methods developed during decades of coral reef ecology research. Overall, it is important to consider the functional definition of recovery and targets for the restoration program, which will be key to implementing a methodology and relevant metrics to construct the baseline dataset of which to monitor the success of an intervention.

**Table 2:** Questions to guide monitoring and research priorities. Questions arising from current knowledge gaps, and examples of ecological and socio-economic metrics to tailor monitoring to the questions for each stage of a rubble field repair intervention.

Question	Ecological metrics	Socio-economic metrics
**Before intervention**		
What are the reasons for the rubble field?	• Nature and history of acute disturbance (eg cyclones, crown-of-thorns starfish, coral bleaching)	• Human use of the area
		• History of human impacts
	• Metrics related to chronic stressors (e.g. turbidity, pollution, ongoing destructive fishing)	
Is the rubble field problematic?	• Repeated measurements of percentage cover of rubble compared to live coral and hard carbonate over time	• Value of the area to fisheries and tourism industries?
		• Importance of intactness and aesthetic appeal?
	• Hydrodynamic properties of the site and rubble movement rates	
	• Count and size of coral recruits	
	• Fish loss	
	• Coral recruit growth and survival	
	• Succession of consolidation and its implication on natural recovery dynamics (Percentage cover of encrusting organisms on rubble; spot sampling of whether rubble pieces are bound or not)	
What are the conditions preventing recovery?	• Wave and current data	• Human use of the area
	• Insufficient coral recruitment	
	• Rubble movement that can be tolerated by coral recruits/juveniles	
	• Sediment loads	
	• Algal cover and herbivore biomass	
	• Larval supply	
**During intervention**		
What will work best?	• Determine ecological objectives and relevant metrics	• Cost and benefit analysis
		• Socio-economic risk assessment
	• Spatial scale	
		• Determine socio-economic objectives and relevant metrics
	• Ecological risk assessment	
**During & after intervention**		
Is the method appropriate?	• Structural integrity of material over time	• Community/visitor concerns/ support/ benefits
	• Changes in rubble movement / consolidation rates	
		• Traditional Owner/Indigenous concerns/ support/ benefits
	• Monitoring of identified risks (e.g. hitchhiking organisms, microbial communities, introduction of foreign material, marine debris)	
	• Introduction and safe storage / isolation of foreign materials	
Is the method working?	• Monitoring tailored to measure metrics relevant to intervention objectives	• Monitoring tailored to measure metrics relevant to intervention objectives
	• Examples: percent cover of rubble vs. consolidated substratum, coral recruitment and recruit survival, coral cover, structural complexity, fish assemblage structure, abundance and biomass.	
		• Examples: aesthetic appeal, tourism and fisheries benefit, cultural significance, community participation
	• Control sites for comparison–both undamaged and unrestored	
	• Coral donor source monitoring if corals transplanted or grown	

**Fig 6:**
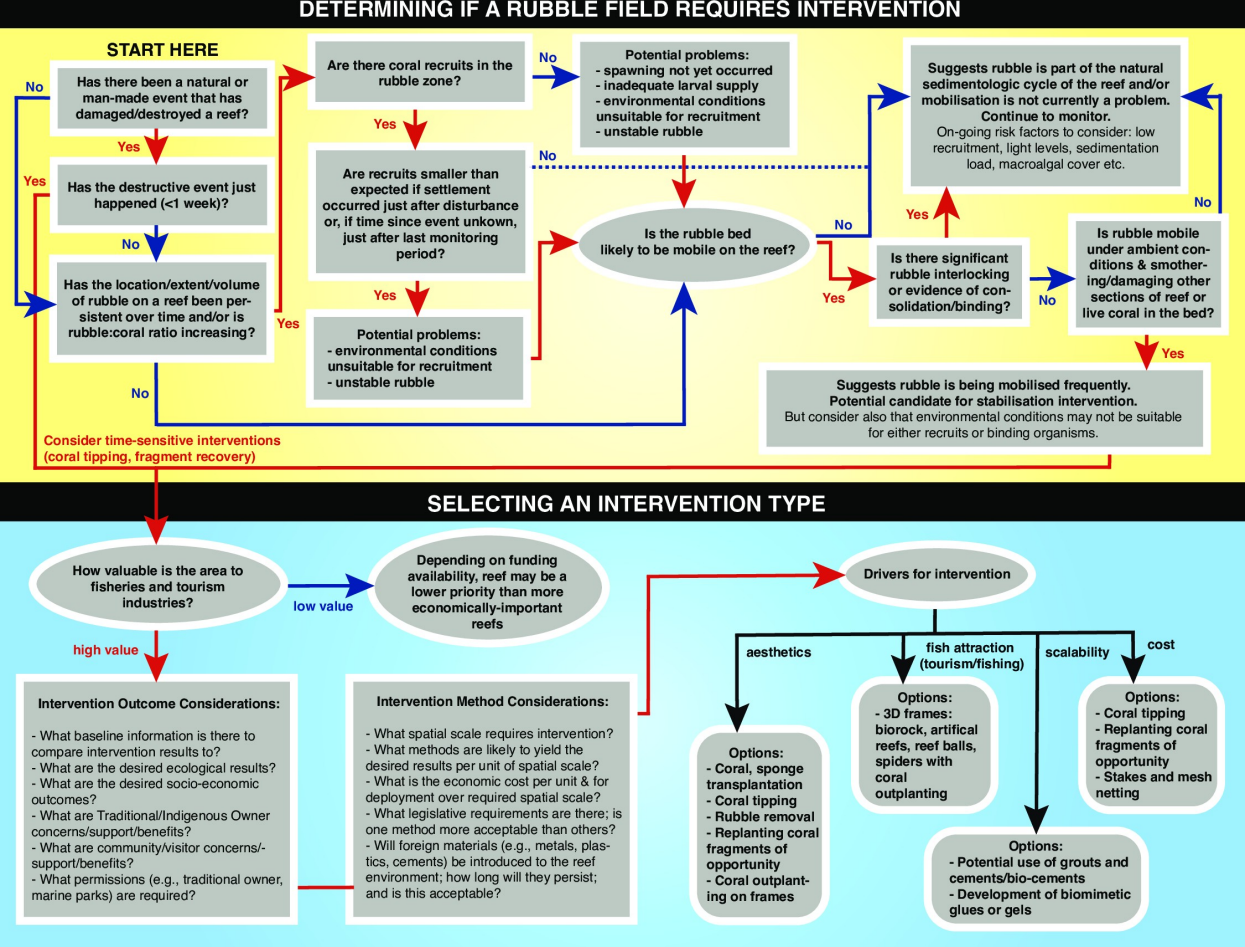
Decision tree showing considerations to be made in rubble stabilisation interventions. The tree shows a framework for making decisions at two stages of restoration planning: when i) determining whether active intervention is suitable and likely to effectively restore a rubble field on a damaged reef, and ii) deciding which active intervention method to employ.

Ideally, data resulting from robust and systematic monitoring should form the foundation of the decision-making process governing if, when, how and what active intervention should be considered for a certain reef (Fig 6). It is also important to consider that the majority of the world’s reefs are located in developing nations, and often in remote locations. Coastal communities in such locations often have little access to human capital that can operate underwater and make educated assessments of the state of rubble, coral recruitment or biological assemblages [129]. Implementing this type of process (Fig 6) may require capacity building in local communities.

Some of the studies reviewed here (Table 1 and S3 Appendix) described the medium-term baseline conditions (e.g. rubble fields that had not recovered for > 6 years, [15, 117]), while others conducted a one-off “baseline” assessment prior to installation of structures [106]. Where pre-intervention monitoring exists, this may set the goals to be achieved during restoration. For example, rubble fields at Havannah Island on the Great Barrier Reef failed to recover for a decade [16, 17], and might therefore be a candidate for intervention. However, before the disturbances, its coral cover was ~45% [16, 17], and some reefs in the same region currently also have cover of ~45% (http://apps.aims.gov.au/reef-monitoring/). Therefore, for restoration at this reef to be considered a success, quantitative and qualitative re-assembly of the coral community should be assessed against historical records and regional norms, which provide benchmarks of the desired ecological state [122]. As always, unmanipulated control sites should also be included at the disturbed reef where no restoration efforts are in place.

Le et al. [130] argued that restoration should be monitored in two distinct phases: 1) the initial establishment phase in which the efficacy of the methods is assessed, and 2) the long-term building phase where monitoring measures performance against ecological objectives (Table 2). If objectives are linked to the recovery of coral ecosystems, then efficacy should be measured for at least five to seven years to include successful recruitment and growth of mature coral colonies, and resistance to potential ongoing disturbances. With a few exceptions [e.g. 69], the monitoring of restoration projects, often linked to student projects or short-term funding, is too brief to ascertain whether the project has resulted in a viable, self-sustaining coral reef community [14]. However, where objectives are stated as simply the enhancement of aesthetic appeal (e.g. a new dive site for tourism) or the remediation of a damaging one-off event (e.g. a ship grounding), a shorter-term monitoring program can be sufficient [106, 131].

Considering the importance of rubble stabilisation, binding and cementation to coral recruitment, it appears essential to not only monitor coral recovery by measuring recruitment and coral cover, but to also assess the degree of stabilisation of a rubble field over time (Table 2). Potential metrics include the thickness of the rubble bank above the underlying substrate, movement of individual rubble pieces in the pile, the height of the rubble pile above the substrate [19, 21] and the force required to shift rubble pieces. Further, knowledge of natural binding times and succession following stabilisation will allow us to more accurately predict recovery times both with and without interventions in place. At a larger scale, an understanding of the hydrodynamics of a reef, together with the size and morphology of the rubble pieces, sediment generation and dispersal across reefs, and the distribution of rubble-encrusting organisms with the potential to bind rubble, should provide an indication of the potential for stabilisation (Fig 6). While further work is conducted to fill these and other knowledge gaps, baseline data on rubble fields could be incorporated into existing reef monitoring programs, including size, morphological categories, reef bathymetry and percentage hard carbonate. Rubble-encrusting organisms such as encrusting sponges and coralline algae could also be added to the small structures, and included in reef monitoring frameworks, and baselines for future monitoring could categorise communities found at different stages of stabilisation succession to estimate degree of stabilisation.

## Conclusions

In environments without direct anthropogenic pressures, natural processes may allow the stabilisation and cementation of coral rubble over time, through a succession of processes that culminates in the return of rubble fields to consolidated reef and living coral assemblages. However, as coral reefs become subject to increasing pressures from stronger and more frequent disturbances producing rubble, the natural capacity of these systems to accrete and generate a consolidated reef framework could be impaired [132, 133], leading to an overall increase in rubble over time. Coral larvae can settle on unstable rubble, but abrasion and smothering of juvenile corals results in negligible survival, such that rubble beds have been dubbed "killing fields" for juvenile corals. We have synthesised the current understanding of the process of natural rubble stabilisation, and the ways in which this knowledge could be used to assess the likelihood that natural stabilisation will occur, or whether there is a need for intervention (Fig 6).

Active interventions may be a way of helping to bridge the gap between the current conditions, the predicted worsening situation, and a hoped-for reprieve in the future, even if at small scales and with primarily aesthetic results. The scale of disturbance-induced rubble lends itself to the scale of common intervention methods. In fact, increasing the speed of recovery is already possible at these small scales, as demonstrated by those studies that have included long-term monitoring. Interventions will likely continue to be especially important at high-value sites. All the methods presented here would ideally be tested with pilot studies and, to reveal whether they work, they will require appropriate monitoring.

Unfortunately, as with most restoration work to date, the methods and techniques reviewed here suffer from a lack of independent monitoring at time scales relevant to the expected outcomes. In fact, the choice of metrics to monitor must be set in relation to the stated objectives of the restoration effort, which are often either absent or vague (e.g. “increasing reef resilience”). If, as the reviewed methods imply, the objectives are to stabilise rubble, promote cementation and create a stable substratum for coral recruitment, then metrics related to rubble stability, cementation rates, and coral settlement and survival are the minimum requirement. We argue that most methods would still require a stage where the efficacy of the technique itself is measured, before including long-term ecological indicators.

Knowledge gaps exist for every stage of the creation, stabilisation and cementation of rubble, the performance of current restoration techniques and the long-term effectiveness of restoration at any scale. In fact, while there are data on the cover of rubble on reefs globally among coral reef monitoring programs, there is a surprising lack of reporting on long-term, large-scale trends in rubble cover. Perhaps the localised nature of both rubble fields created by human disturbance and the current techniques available to restore them makes this metric obsolete at larger scales, and the focus should remain on restoring local sites that are aesthetically, economically or socio-culturally valuable. Coral restoration projects are increasingly including socio-cultural and socio-economic objectives to ensure the long-term sustainability of the restoration efforts. Active involvement in coral restoration activities has been shown to improve opportunities for education and stewardship of reef resources. As accounting for the human dimension of coral restoration efforts becomes more important, monitoring plans should increasingly evaluate metrics such as reef-user satisfaction, stewardship, capacity-building, and economic value. However, active interventions such as substrate stabilisation should be considered as an addition to, and not a substitute for, global action on climate change, conventional management and reducing local and regional stressors.

## Supporting information

S1 Appendix Semi-structured interview for coral tipping and rubble stabilisation scoping study. Interview questions.
(DOCX)

S2 Appendix Natural stabilisation of rubble on coral reefs. (DOCX)

S3 Appendix Case studies of structures and stabilisation techniques. (DOCX)

S4 Appendix Legal considerations. (DOCX)
