# Supplementary material for: Substrate stabilisation and small structures in coral restoration: State of knowledge, and considerations for management and implementation
Source: PLoS One. 2020 Oct 27;15(10):e0240846. doi: 10.1371/journal.pone.0240846 (PMC7591095; doi:10.1371/journal.pone.0240846)
Supplement: S1 Appendix — Interview questions. (DOCX) [file pone.0240846.s001.docx]

Small structures and substrate stabilisation in coral restoration: state of knowledge, and considerations for management and implementation

Daniela M. Ceccarelli, Ian M. McLeod, Lisa Boström-Einarsson, Scott E. Bryan, Kathryn M. Chartrand, Michael J. Emslie, Mark T. Gibbs, Manuel Gonzalez Rivero, Margaux Y. Hein, Andrew Heyward, Tania M. Kenyon, Brett M. Lewis, Neil Mattocks, Maxine Newlands, Marie-Lise Schläppy, David J. Suggett, Line K. Bay

# S1 Appendix

## Semi-Structured Interview for coral tipping and rubble stabilisation scoping study

Ethics ID H7799- Human Research Ethics Committee- James Cook University

*Turn recorder ON*

Hi xx, thank you very much for agreeing to this interview. My name is xx and I’m a researcher at James Cook University in Australia. This interview is part of a project in which we’re looking at the work that is being done around rapid responses on coral reefs after acute disturbances.

Before I start with the questions, for ethics purposes, I just want to confirm that you understand the purpose of this interview, and that you agree to be recorded as part of this process.

*Wait for answer*

Your responses and contact details will be kept strictly confidential within our small research team

Do you have any questions prior to starting?

*Wait for answer*

### Section 1: Demographics and background

**1.** Could you please give me a little bit of background about yourself? Who you are, what you do?

*Wait for answer*

### Section 2: Rapid response strategy

**2**. What kind of impact have you/your team responded to where emergency response was necessary?

*Wait for answer*

**3.** What kind of emergency restoration technique did you apply?

*Wait for answer then select questions from the list below:*

**3.1.** Re- attachment and re-orientation of corals

- What was your re-attachment strategy?

Why?

*Wait for answer*

- Were you focused on specific types of corals?

If yes- which ones and why?

*Wait for answer*

*If cement/Plaster of Paris/ Epoxy was used:*

- Where did you mix it?

*Wait for answer*

- How did you transport it under-water?

*Wait for answer*

- How did you lay it down on the substrate?

*Wait for answer*

**3.2**. Substrate stabilisation

- What kind of method did you use?

Why? *Prompts for details*

*Wait for answer*

- Was it for the sole purpose of stabilisation or was it also used as a surface for coral re-attachment?

*Wait for answer*

**3.3.** Fragments of opportunities

- Did you collect dislodged fragments?

*Wait for answer*

- Were these ‘replanted’?

*Wait for answer*

- How? (refer back to cement/plaster of Paris/epoxy questions in 3.1 if necessary)

*Wait for answer*

- Did you hold them in a nursery before replanting?

*Wait for answer*

If yes:

- What kind of nursery?

*Wait for answer*

- How long did you hold them before replanting?

*Wait for answer*

**3.4** Other

- How did you proceed?

*Prompts:* get as much details as possible on the techniques

*Wait for answer*

### Section 3: Logistics

***Workforce and costs***

**4.** How many people were involved?

*Wait for answer*

**5.** What background skills or training did the workforce have?

*Wait for answer*

*Prompt:* Was there any special preparation prior to the disturbance? Any special plan for rapid response?

**6**. Can you give me a rough estimate of the costs associated with the intervention?

*Wait for answer*

***Timing of response***

**7.** How quickly after the disturbance were you able to respond?

*Wait for answer*

**8.** How did you get approval to do this work?

*Wait for answer*

**9.** How long did it take?

*Wait for answer*

**10.** Was it a one-time off intervention or was it repeated over time?

*Wait for answer*

### Section 4: Lessons learned and recommendations

**11.** Did you revisit the site and monitor success?

*Wait for answer*

*If yes*

*- When? At what frequency?*

- *How did you mark your corals and site(s)?*

*Wait for answer*

*If no*

*-why?*

*Wait for answer*

**12.** Did you notice macro-algae overgrowth at the disturbance site?

*Wait for answer*

*If yes*

- Did you attempt to control it? How?

*Wait for answer*

**13.** Would you say your effort was successful?

*Wait for answer*

Why? *Prompt for their idea of success and failure*

**14**. With unlimited budget and time, what would you have liked to do differently?

*Prompt:* What were your barriers: Cost, time, number of people, type of people, permits etc..

*Wait for answer*

**15.** What would you say are the most important factors to consider about xx rapid response type of intervention?

*Wait for answer*

Ok xx. I think we have covered everything. Is there anything else you would like to tell me that I haven’t covered?

*Wait for answer*

Wonderful. Thank you again for your time and insights. Don’t hesitate to get in touch if you have any more questions or thoughts on this subject.

*Turn recorder OFF*
