# Supplementary material for: Substrate stabilisation and small structures in coral restoration: State of knowledge, and considerations for management and implementation
Source: PLoS One. 2020 Oct 27;15(10):e0240846. doi: 10.1371/journal.pone.0240846 (PMC7591095; doi:10.1371/journal.pone.0240846)
Supplement: S2 Appendix — (DOCX) [file pone.0240846.s002.docx]

Small structures and substrate stabilisation in coral restoration: state of knowledge, and considerations for management and implementation

Daniela M. Ceccarelli, Ian M. McLeod, Lisa Boström-Einarsson, Scott E. Bryan, Kathryn M. Chartrand, Michael J. Emslie, Mark T. Gibbs, Manuel Gonzalez Rivero, Margaux Y. Hein, Andrew Heyward, Tania M. Kenyon, Brett M. Lewis, Neil Mattocks, Maxine Newlands, Marie-Lise Schläppy, David J. Suggett, Line K. Bay

# S2 Appendix

## Natural stabilisation of rubble on coral reefs

Depending on its location across the reef environment, rubble may be transported onshore or offshore, or may become trapped within the reef matrix. If rubble remains stable for long enough, it can eventually become consolidated and incorporated into the reef framework [1, 2]. The transition from rubble to reef has been described as a two-part process that requires both a preliminary stage of rubble stabilization and binding, followed by rigid binding or diagenetic cementation [2]. The processes of rubble stabilisation, binding and cementation increases reef density and decreases reef porosity, leading to reduced erodibility and increased stabilisation of the substrate [3]. Even though this is a critical process in the recovery of damaged coral reef communities, qualitative analysis and understanding of reef framework and rubble stabilisation and binding rates is limited [3].

### Preliminary stabilisation and temporary binding

Preliminary stabilisation of rubble can be achieved when hydrodynamic energy is reduced or rubble pieces interlock (Main Document, Fig 2a)***,*** followed by the colonisation of encrusting and binding invertebrates (Main Document, Fig 2b, c)[2, 4, 5]. Organisms that encrust and bind rubble include macroalgae, sponges, crustose coralline algae, corals, corallimorpharians, colonial bryozoans, colonial ascidians, zooanthids, serpulid worms, hydrozoans and foraminifera [5-11]. Dependant on the environmental conditions and recruitment potential, rubble-encrusting organisms colonise rubble at different times and at different rates. The distribution and growth of these organisms is influenced by environmental factors including light availability, hydrodynamic regime and sedimentation [2, 12-14]. For example, photosynthetic organisms such as turf algae, macroalgae and coralline algae are more prevalent in shallower areas on exposed surfaces with high light availability [15-17], and coralline algae are more prevalent in high energy environments, due to their low profile [18].

Inferences about the order of colonisation of rubble binding organisms can be made from studies of succession on recruitment tiles. Turf algae are often the first colonisers during early succession [19], increasing rapidly over the first ~3 months [20]. This is usually followed by encrusting and fleshy macroalgae, and coralline algae begin to dominate after ~6 months [20]. Macroalgae can occupy space rapidly, particularly after events that reduce coral cover [21]. In the Caribbean, macroalgae (primarily *Dictyota, Caulerpa*, and *Halimeda spp.*) grew over and bound 62% of experimental rubble piles securely after one month [5]. However, macroalgae is often highly seasonal [22, 23], and therefore may only bind rubble temporarily [5]. If high algal cover persists (for example, because herbivory levels are low) it can inhibit the recruitment and growth of stronger binders like crustose coralline algae and corals.

### Rigid binding

Following preliminary stabilisation, rigid binding by laterally-growing carbonate encrusting organisms and diagenetic cementation can occur (Main Document, Fig 2d). Providing herbivory levels are adequate to control turf and macroalgae, the cover of colonial bryozoans, ascidians and sponges has been shown to increase on recruitment tiles after ~3 months [20]. Sponges are considered critical for the initial stabilisation and binding of coral rubble [2, 5, 24, 25], but there is little research on how quickly sponges bind rubble. In the Caribbean, cryptic sponges adhered to the underside of more than 50% of experimental rubble piles within one month, and had tightly bound 73% of piles after five months [5].

Other studies that seeded experimental rubble piles with erect sponge species found that preliminary stabilisation and binding to the point where individual rubble pieces were not distinguishable took between ten [5] and 12 months [8]. Other soft-bodied invertebrates, including zooanthids and corallimorphs, can also bind rubble piles [8; T. Kenyon, pers. obs., 11]. However, the potential of corallimorphs to aid recovery may be negated by their ability to outcompete other taxa. In the Maldives they covered 80% of the benthos at some islands and overgrew coralline algae, sponges and reduced space for coral settlement [11].

### Carbonate binders

Carbonate encrusters like colonial bryozoans, which are highly successful in cryptic microhabitats [12, 26], are important binders and have been known to outcompete other colonisers including foraminiferans, bivalves and serpulid worms in rubble beds [27]. Crustose coralline algae are also considered by several studies to be strong and important binders due to their encrusting, calcified structure and resilience to high wave exposure [2, 7, 28]. They have also been reported to encrust and firmly bind experimental, sponge-seeded rubble piles after just seven months [5]. However, experimental rubble piles that were coralline algae-encrusted, but not previously seeded with sponges, remained un-consolidated [5]. This suggests that effective carbonate binding may require a combination of different organisms. ‘Algal cup reef’ structures, for example, are characterized by vertical layering of not only coralline algae, but also encrusting corals, foraminifera and vermetid gastropods [29]. On a larger scale, coralline algal crusts have formed over the top of vast coral rubble deposits on fringing reefs in the Caribbean following hurricanes [1], though the time-frame for this process is unknown. In some cases, crusts are present in some areas but the rubble ridge remains an unconsolidated, loose pile after 3 years [30]. Even when consolidation has occurred, and algal crusts provide a firm substrate for lateral regeneration of the reef, the rubble beneath the veneer might remain unconsolidated [7], necessitating in-filling and cementation.

### Cementation

Following preliminary stabilisation and binding, rigid binding by diagenetic cementation occurs in near-shore ramparts as well as back-reef, reef-flat, and shallow fore-reef rubble patches [1, 2, 31]. After rubble is initially deposited and reef framework is formed by corals and other marine calcifiers, the reef matrix undergoes diagenetic alteration and is continuously infilled with sediment, rubble, and cements [2, 32, 33]. These cements are largely composed of high‐magnesium calcite and aragonite that infill the voids between the rubble pieces and encrust over the dead skeletal materials [1, 2, 33]. Calcifying microbialites further trap and bind sediments and are important in rigid cementation. Other encrusters that are also preliminary binders, such as coralline algae, corals and bryozoans, are of secondary importance at this stage [2]. The lithification of coral rubble is a critical diagenetic process that leads to rigid substrate cementation [31, 32]. The degree of rubble cementation is considered a function of time available at the interface of the water column and the rubble substratum. Thus, slower accreting reefs are more consolidated and cemented than those with higher accretion rates [2]. The highest rates of rigid rubble cementation are found in fore-reef areas with low sloping angles above the wave base, while the lowest rates are found in deeper fore-reef environments and the reef crest [2].

# References

1. Blanchon P, Richards S, Bernal JP, Cerdeira-Estrada S, Ibarra M, Corona-Martínez L, Martell-Dubois R. Retrograde accretion of a Caribbean fringing reef controlled by hurricanes and sea-level rise. Frontiers in Earth Science. 2017;5:78.

2. Rasser MW, Riegl B. Holocene coral reef rubble and its binding agents. Coral Reefs. 2002;21:57-72.

3. Kuffner IB, Toth LT. A geological perspective on the degradation and conservation of western Atlantic coral reefs. Conservation Biology. 2016;30:706-15.

4. Cameron CM, Pausch RE, Miller MW. Coral recruitment dynamics and substrate mobility in a rubble-dominated back reef habitat. Bulletin of Marine Science. 2016;92:123-36.

5. Wulff J. Sponge-mediated coral reef growth and rejuvenation. Coral Reefs. 1984;3:157-63.

6. Perrin C. Solenomeris: From biomineralization patterns to diagenesis. Facies. 2009;55:501-22.

7. Minnery GA. Crustose coralline algae from the Flower Garden Banks, Northwestern Gulf of Mexico: controls on distribution and growth morphology. Journal of Sedimentary Petrology. 1990;60:992-1007.

8. Biggs BC. Harnessing natural recovery processes to improve restoration outcomes: an experimental assessment of sponge-mediated coral reef restoration. PLoS ONE. 2013;8:1–15. doi: ARTN e64945 10.1371/journal.pone.0064945.

9. Alexandersson ET. Carbonate cementation in recent coralline algal constructions. In: Flugel E, editor. Fossil algae. New York: Springer Berlin Heidelberg; 1974. p. 261–9.

10. Rojas PTJ, Raymundo LJ, Myers RL, editors. Coral transplants as rubble stabilizers: a technique to rehabilitate damaged reefs. Proceedings of the 11th International Coral Reef Symposium; 2008.

11. Zahir H, Quinn N, Cargilia N. Assessment of Maldivian coral reefs in 2009 after natural disasters. Male, Maldives: Marine Research Centre, Ministry of Fisheries Agriculture and Marine Resources, 2009.

12. Baird AH, Hughes TP. Competitive dominance by tabular corals: an experimental analysis of recruitment and survival of understorey assemblages. Journal of Experimental Marine Biology and Ecology. 2000;251:117-32.

13. Fabricius K, De’ath G. Environmental factors associated with the spatial distribution of crustose coralline algae on the Great Barrier Reef. Coral Reefs. 2001;19:303-9.

14. Mallela J. Coral reef encruster communities and carbonate production in cryptic and exposed coral reef habitats along a gradient of terrestrial disturbance. Coral Reefs. 2007;26:775-85.

15. Martindale W. Calcified epibionts as palaeoecological tools: examples from the Recent and Pleistocene reefs of Barbados. Coral Reefs. 1992;11:167-77.

16. Dean AJ, Steneck RS, Tager D, Pandolfi JM. Distribution, abundance and diversity of crustose coralline algaeon the Great Barrier Reef. Coral Reefs. 2015:DOI 10.1007/s00338-015-1263-5.

17. Morgan KM, Kench PS. Reef to island sediment connections on a Maldivian carbonate platform: using benthicecology and biosedimentary depositional facies toexamine island-building potential. Earth Surface Processes and Landforms. 2016:DOI: 10.1002/esp.3946.

18. Steneck RS, Dethier M. A functional group approach to the structure of algal-dominated communities. Oikos. 1994;69:DOI: 10.2307/3545860.

19. McClanahan TR. Primary succession of coral-reef algae: Differing patterns on fished versus unfished reefs. Journal of Experimental Marine Biology and Ecology. 1997;218:77-102.

20. Doropoulos C, Roff G, Bozec Y-M, Zupan M, Werminghausen J, Mumby PJ. Characterizing the ecological trade‐offs throughout the early ontogeny of coral recruitment. Ecological Monographs. 2016;86:20-44.

21. Mumby PJ, Foster NL, Fahy EAG. Patch dynamics of coral reef macroalgae under chronic and acute disturbance. Coral Reefs. 2005;24:681-92.

22. Diaz-Pulido G, McCook LJ, Dove S, Berkelmans R, Roff G, Kline DI, Weeks S, Evans RD, Williamson DH, Hoegh-Guldberg O. Doom and boom on a resilient reef: Climate change, algal overgrowth and coral recovery. Public Library of Science. 2009;4:1-9.

23. Mumby PJ. Herbivory versus corallivory: Are parrotfish good or bad for Caribbean coral reefs? Coral Reefs. 2009;28:683-90.

24. Ávila E, Riosmena-Rodríguez R, Hinojosa-Arango G. Sponge–rhodolith interactions in a subtropical estuarine system. Helgoland Marine Research. 2013;67:349.

25. Scoffin TP, Hendry MD. Shallow-water sclerosponges on Jamaican reefs and a criterion for recognition of hurricane deposits. Nature. 1984;307:728-9.

26. Jackson JBC. Overgrowth competition between encrusting cheilostome ectoprocts in a Jamaican cryptic reef environment Journal of Animal Ecology. 1979;48:805-23.

27. Choi DR. Ecological succession of reef cavity-dwellers (coelobites) in coral rubble. Bulletin of Marine Science. 1984;35:72-9.

28. Bosence DWJ. The “coralligene” of the Mediterranean – a recent analog for the tertiary coralline algal limestones. In: Toomey DE, Nitecki MH, editors. Paleoalgology: Contemporary Research and Applications. Berlin: Springer; 1985. p. 216-25.

29. Ginsburg RN, Schroeder JH. Growth and submarine fossilization of algal cup reefs, Bermuda. Sedimentology. 1973;20:575-614.

30. Scoffin TP, McLean RF. Exposed limestones of the northern province of the Great Barrier Reef. Philosophical Transactions of the Royal Society London. 1978;291:119-38.

31. Perry CT, Alvarez‐Filip L. Changing geo‐ecological functions of coral reefs in the Anthropocene. Functional Ecology. 2018;33:976-88.

32. Perry CT, Hepburn LJ. Syn-depositional alteration of coral reef framework through bioerosion, encrustation and cementation: Taphonomic signatures of reef accretion and reef depositional events. Earth-Science Reviews. 2008;86:106-44.

33. Beltrán Y, Cerqueda-García D, Tas N, Thomé PE, Iglesias-Prieto R, Falcón LI. Microbial composition of biofilms associated with lithifying rubble of *Acropora palmata* branches. FEMS Microbiology and Ecology. 2016;92:1-10.
