# Supplementary material for: Substrate stabilisation and small structures in coral restoration: State of knowledge, and considerations for management and implementation
Source: PLoS One. 2020 Oct 27;15(10):e0240846. doi: 10.1371/journal.pone.0240846 (PMC7591095; doi:10.1371/journal.pone.0240846)
Supplement: S3 Appendix — (DOCX) [file pone.0240846.s003.docx]

Small structures and substrate stabilisation in coral restoration: state of knowledge, and considerations for management and implementation

Daniela M. Ceccarelli, Ian M. McLeod, Lisa Boström-Einarsson, Scott E. Bryan, Kathryn M. Chartrand, Michael J. Emslie, Mark T. Gibbs, Manuel Gonzalez Rivero, Margaux Y. Hein, Andrew Heyward, Tania M. Kenyon, Brett M. Lewis, Neil Mattocks, Maxine Newlands, Marie-Lise Schläppy, David J. Suggett, Line K. Bay

# S3 Appendix

## Case studies of structures and stabilisation techniques

### MARRS ‘Reef Stars’

In recent years, Mars Symbioscience, a commercial entity of Mars Incorporated, has developed a modular small structure (the “Reef Star”) for the purposes of coral restoration on unstable or soft substrata. The stars were developed in response to acute and localised disturbances on South East Asian reefs from blast fishing, coastal development and coral mining. The structures are small, open, hexagonal structures resembling a six-legged “star”, fabricated from six metres of 10mm diameter steel bar coated in a protective coating of resin and coarse sand. The stars are staked into the substrate, creating a robust matrix of structures with capacity to withstand wave activity and storms. The modular units are tied together to form a ‘web’ to fill gaps between remnant live coral patches, while stabilising coral rubble in degraded patches. Coral fragments sourced from nearby healthy reefs as ‘corals of opportunity’ (coral fragments already dislodged from their parent colonies by disturbance) are attached to the structures using cable ties. Over time, the structures are incorporated into the reef structure matrix as coral fragments grow. Depending on the populations of herbivores present at the restoration site, some maintenance may be required for the first three months, including macroalgal removal until fish grazing and other natural processes take over. More than 11,000 stars have been deployed over 7000 m^2^ of severely degraded reefs surrounding Pulau Badi island in Indonesia. Early results describe an increase in coral cover from 10% to over 50% on some sites after three years [1]. Critically, the projects in Indonesia have been combined with traditional management like the establishment of no-take marine reserves [1].

### Rock piles

In Komodo National Park, Indonesia, large rubble fields created by chronic dynamite fishing and coral mining showed no signs of recovery over a 6-year period after disturbances ceased in the late 1990s [2, 3]. In 2002, quarried rocks (mean length: 20-30 cm) were introduced to stabilise and add structure to the rubble fields. Rock piles were deployed at four sites that varied in current flow velocity (there was no replication of site type). The piles, each with approximately 140 m^3^ of rock, were arranged in four different configurations: (i) a single, continuous rock pile; (ii) 4-8 rock piles parallel to the prevailing currents; (iii) rock piles perpendicular to the prevailing currents; and (iv) many (>20) rock piles of 1-2 m^3^ [4]. Benthic cover was monitored in restored areas (on the rocks) and control rubble fields prior to the restoration effort from 1998-2000, and afterwards in 2004, 2008 and 2016 [5].

Across configuration treatments, hard coral cover increased from 0% in 2002 to 44.5% (±21.9% SD) in 2016, while hard coral cover in control, non-rehabilitated rubble fields remained at approximately 3%. While the effect of increasing coral cover was generally similar across rock pile configurations, the perpendicular (iii) and multiple (iv) rock piles showed the greatest increase in coral cover, the continuous rock pile (i) configuration had the lowest coral cover (3.2%), and the highest overall increase in coral cover (82.5%) was observed on the parallel pile at the site with comparatively lower flow. The lack of replication makes it difficult to determine the importance of site (and associated variation in flow) and configuration in driving these differences. Nevertheless, the authors report a trend in which rehabilitation was most effective in areas of moderate flow, regardless of configuration type. The cost of this project was: USD$33,000, or USD$5/m^2^.

### Plastic mesh, rock piles and metal stakes

This case study demonstrates a combination of rubble stabilisation techniques and deployment of small structures [6]. Dynamite fishing in Negros Oriental, Philippines, resulted in large rubble fields that showed little recovery, even after the cessation of destructive fishing in the 1980s and establishment of the Calagcalag Marine Protected Area in 1988. In 2003, within the 2,400 m^2^ rubble field on the reef flat, five 17.5 m^2^ plots were rehabilitated – three in June during the spawning season, and two in October. The rehabilitation consisted of pinning down the rubble with 2 cm plastic mesh, anchoring the mesh with rebar stakes, and adding rock piles on top. Rock piles were hollow, made of reef rock and cement, and were pyramid shaped (0.5 m^2^ at the base and 1 m in height). ﻿Fish recruitment (abundance and length) and coral recruitment (abundance and length, including of tagged recruits) were monitored three to four times per year in the rehabilitation plots and at adjacent un-rehabilitated rubble and ‘healthy’ reef. Benthic composition was measured in 2003 as a baseline assessment and in 2005 in the rehabilitated area (including plots and the space in-between), un-rehabilitated rubble and adjacent reef.

After a three-year period, both the rehabilitated plots and adjacent reef had significantly higher fish biomass compared to the un-rehabilitated rubble area. ﻿Coral recruit abundance increased in the rehabilitated plots deployed during the spawning season from an average of 0.5 ind. m^-2^ after two months to 4.5 ind. m^-2^ after two years. Unfortunately, the study does not specify the number of recruits per m^2^ in unrehabilitated rubble. The survival of tagged coral recruits was significantly higher in the rehabilitated plots (63.4% ± 32% in 10 months) compared to un-rehabilitated rubble (6% ± 10% for the same time period), and recruits in the rehabilitated plots grew larger. Recruits on un-rehabilitated rubble were generally abraded and most died, disappeared or remained very small (2-4 cm in diameter) over the monitoring period. The increase in coral cover in the rehabilitated plots was higher than in unrehabilitated rubble, but not significantly so. As both small structures and substrate stabilisation were utilised in this study, it is difficult to ascertain the most effective driver of increased fish and coral recruitment. However, corals were observed to grow on both the structures and netting. The cost of this project was ﻿approximately USD$4.30 m^-2^ of rehabilitated plot. ﻿If the entire 2,400 m^2^ rubble field was rehabilitated, the cost would be USD$10,560. However, if the space between the plots is also considered as rehabilitated area, and rehabilitation plots are established at a ratio of approximately five per 500 m^2^, the cost would be reduced to approximately USD$1,800 or USD$0.75 m^-2^.

### Rubble removal

Where rubble stabilisation is logistically or financially not possible, removing loose rubble from the impacted site might be the first course of action. The scale of removal can vary from a few kilograms of rubble removed by hand and put in bags or bins to hundreds of metric tonnes removed using suction tubes. In most cases, rubble is then disposed to in deeper areas. In Hawai’i, over 700 metric tonnes of rubble were removed from a site impacted by a large ship grounding [7]. A specifically designed suction tube was hooked to a compressor on the boat, the rubble was stocked in large bins on the boat and moved offshore. The method was successful after a number of engineering attempts to determine the size of the hose with respect to the size of the rubble pieces (M. Perry, NOAA, pers. comm.).

## References

1. Williams SL, Sur C, Janetski N, Hollarsmith JA, Rapi S, Barron L, Heatwole SJ, Yusuf AM, Yusuf S, Jompa J, Mars F. Large-scale coral reef rehabilitation after blast fishing in Indonesia. Restoration Ecology. 2019;27:447-56.

2. Fox HE, Caldwell RL. Recovery from blast fishing on coral reefs: A tale of two scales. Ecological Applications. 2006;16:1631–5.

3. Fox HE, Pet JS, Dahuri R, Caldwell RL. Recovery in rubble fields: long-term impacts of blast fishing. Marine Pollution Bulletin. 2003;46(8):1024-31.

4. Fox HE, Mous PJ, Pet JS, Muljadi AH, Caldwell RL. Experimental assessment of coral reef rehabilitation following blast fishing. Conservation Biology. 2005;19:98-107.

5. Fox HE, Harris JL, Darling ES, Ahmadia GN, Razak TB. Rebuilding coral reefs: success (and failure) 16 years after low-cost, low-tech restoration. Restoration Ecology. 2019;27:862-9.

6. Raymundo LJ, Maypa AP, Gomez ED, Cadiz P. Can dynamite-blasted reefs recover? A novel, low-tech approach to stimulating natural recovery in fish and coral populations. Marine Pollution Bulletin. 2007;54:1009-19.

7. NOAA, DLNR. Final damage assessment and restoration plan and NEPA evaluation for the February 5, 2010, M/V Vogetrader grounding at Kalaeloa, Barbers Point, Oahu. Hawai’i: National Oceanic and Atmospheric Administration and Department of Land and Natural Resouces, 2017.
