# Supplementary material for: Substrate stabilisation and small structures in coral restoration: State of knowledge, and considerations for management and implementation
Source: PLoS One. 2020 Oct 27;15(10):e0240846. doi: 10.1371/journal.pone.0240846 (PMC7591095; doi:10.1371/journal.pone.0240846)
Supplement: S4 Appendix — (DOCX) [file pone.0240846.s004.docx]

Small structures and substrate stabilisation in coral restoration: state of knowledge, and considerations for management and implementation

Daniela M. Ceccarelli, Ian M. McLeod, Lisa Boström-Einarsson, Scott E. Bryan, Kathryn M. Chartrand, Michael J. Emslie, Mark T. Gibbs, Manuel Gonzalez Rivero, Margaux Y. Hein, Andrew Heyward, Tania M. Kenyon, Brett M. Lewis, Neil Mattocks, Maxine Newlands, Marie-Lise Schläppy, David J. Suggett, Line K. Bay

# S4 Appendix

## Legal considerations

The installation of structures for reef restoration, given some of the potential risks and multiple and overlapping uses of marine areas, both protected and unprotected, can require complex approval processes. The rigour of the approval process for specific restoration projects will also generally be dependent on the level of risk and location. This may relate to the size of the structures and the materials used, as well as site-specific matters such as marine park zoning, levels and types of current recreational and commercial use of the area and existing habitat condition. This degree of complexity, and the standards of information required in any approvals process, will also depend on the specific country and its relevant legislation and policies. For example, in Australia even small structures come under the legislation for 'artificial reefs', which have associated guidelines developed to manage their installation under the Convention on the Prevention of Marine Pollution by Dumping of Wastes and Other Matter 1972, or the London Convention. Today the more modern version the London Protocol (1996) remains relevant to all member states of the United Nations Environment Program [1]. Depending on the nature of the reef restoration project and materials being used, it may be considered by the governing authority administering the guidelines that some form of ‘artificial reef’ approval is required.

## Regulatory Case Study: Australia and Underwater Structures

Placing structures or “artificial reefs” into Australian waters is regulated under the federal government’s *Environment Protection (Sea Dumping) Act* 1981 (Commonwealth). The Act allows for the placement of structures, including artificial reefs, in the marine environment under the Commonwealth jurisdiction. However, any permissions will include evaluating the level of risk and impact any artificial reef may have on a marine park, fisheries resources, habitat and marine plants [2]. In Australia, small artificial reefs (=<20 m²) are assessed under the federal Environment Protection and Biodiversity Conservation Act (EPBC), the Environment Protection (Sea Dumping) Act 1981, or the Sea Installations Act 1987. In 2019, the cost of assessing a Sea Dumping Act application was AU$10,000, regardless of the size of the project or if the objective of the project was for restoration and environmental improvement. The EPBC Act also sufficiently covers Australia’s commitment to the London Protocol. Additionally, the United Nations Framework Convention on Climate Change (UNFCCC) Paris Accord stipulates reducing pressures on corals from warming waters and ocean acidification, which may be interpreted to include active interventions where these impacts have already caused damage.

Engaging with managing authorities *before* the need for a specific action is advisable, preferably concurrent to the project planning stages. For example, the assessment costs for Sea Dumping Act permits can be waived for certain projects. The success of some interventions may rely on rapid responses after a significant impact, but even in these situations ‘pre-approval’ to undertake the action may be possible. Depending on the size of the project there may also be requirements to undertake comprehensive public consultation, post financial bonds (to guarantee site clean-up if needed), provide insurance and engage in specific contractual arrangements with governing bodies. Ongoing engagement between all stakeholders (government, researchers, industry, indigenous and community groups) as concepts and practices move forward should allow for more rapid consideration of any approvals required.

**Literature cited**

1. IMO. Convention on the prevention of marine pollution by dumping of wastes and other matter 2019 [Accessed 8 December 2019]. Available from: <http://www.imo.org/en/OurWork/Environment/LCLP/Pages/default.aspx>.

2. Fidelman P, McGrath C, Newlands M, Dobbs K, Jago B, Hussey K. Regulatory implications of coral reef restoration and adaptation under a changing climate. Environmental Science & Policy. 2019;100:221-9.
